# Effects of thoracic paravertebral nerve block on postoperative pain and postoperative delirium in elderly patients undergoing thoracoscopic lobectomy

**DOI:** 10.1097/MD.0000000000032907

**Published:** 2023-02-22

**Authors:** Qiu Dongjie, Zhao Longbiao, Liu Peng, Jia Li, Xu Hongmeng, Chang Zhiyan, Yu Long

**Affiliations:** a Department of Anesthesiology, The Fourth Hospital of Hebei Medical University, Shijiazhuang, PR China; b Department of Anesthesiology, The Third Hospital of Hebei Medical University, Shijiazhuang, PR China.

**Keywords:** elderly patient, pain, postoperative delirium, thoracic paravertebral block, ultrasound

## Abstract

**Objectives:**

To evaluate the effects of ultrasound-guided thoracic paravertebral nerve block on perioperative pain and postoperative delirium in elderly patients undergoing thoracoscopic lobotomy.

**Methods:**

Patients aged 60 to 80 years who underwent the surgery of thoracoscopic lobectomy were selected; ASA grades I to III and New York Heart Association (NYHA) grades I to II. Patients were randomly divided into two groups: group C (group Compaired) and group T (group Thoracic Paravertebral Nerve Block TPVB). Patients in group T received ultrason-guided TPVB while those in group C didn’t received TPVB. Postoperative patient-controlled intravenous analgesia was administered to all the patients. The consumption of intraoperative opioids, cases of hipoxemia, operative time, and extubation time was also recorded. Pain scores (static and dynamic) were assessed at 2, 4, 6, 24, 48, 72, 96, and 120 hours point after the operation. Pain scores, occurrence of postoperative delirium occurrence, postoperative complications, total amount of analgesic drugs, length of hospital stay, rescue analgesic requirement, and side effects were recorded within 5 days.

**Results:**

Intraoperative dosages of sufentanil and remifentanil were significantly lower in group T (Table 1). The postoperative recovery time in group T was significantly shortened (Table 1). The VAS pain scores of group T at 2, 4, 6, and 24 hours after surgery were much lower. The consumption of intraoperative opioids, number of rescue analgesic requirements, and the occurrence of postoperative delirium incidence in group T was significantly reduced (Table 2). There were no differences in hipoxemia events, postoperative nausea, vomiting and pulmonary complications between the two groups (Table 2).

**Conclusion:**

Preoperative ultrasound-guided thoracic paravertebral nerve block (TPVB) can obviously decrease the intraoperative and postoperative opioids consumption, shorten the recovery time, reduce the number of rescue analgesia and the incidence of postoperative delirium in elderly patients undergoing thoracoscopic lobotomy.

**Table 1:** Sufentanil and remifentanil consumption.

The total amount of	Group C (n = 105)	Group T (n = 103)	*P* value
Sufentanil (µg/kg)	0.91 ± 0.26	0.68 ± 0.17**	<.01
Remifentanil (µg/kg)	18.8 ± 2.9	14.7 ± 1.5**	<.01

Values are expressed as means ± SD.

*P < .05.

** *P* < .01.

**Table 2:** Postoperative static VAS pain score.

	2 h	4 h	6 h	24 h	48 h	72 h	96 h	120 h
Group C (n = 105)	3.6 ± 1.1	3.7 ± 1.0	2.9 ± 0.9	2.6 ± 1.3	1.4 ± 0.8	0.6 ± 0.6	0.1 ± 0.3	0
Group T (n = 103)	2.6 ± 0.9**	2.5 ± 0.8**	2.1 ± 0.7**	1.7 ± 0.7**	1.2 ± 0.8	0.7 ± 0.7	0 ± 0.22	0
*P*	<.01	<.01	<.01	<.01	.129	.571	.128	

*P < .05.

** *P* < .01.

## 1. Introduction

Video-assisted thoracoscopic surgery (VATS) has become increasingly popular because of its faster recovery time and less postoperative complications.^[1]^ Despite its association with reduced surgical trauma, better postoperative outcome, and decreased tissue injury VATS does not necessarily lead to reduced analgesia requirement in the same extent.^[2]^ In recent years, thoracic paraspinal nerve block (TPVB) has been widely used in the perioperative period of thoracic surgery. Ultrasound-guided thoracic nerve block can further improve the accuracy and safety.^[3]^ Enhanced recovery after surgery advocates perioperative multimodal analgesia, reduces opioid use and promotes rapid recovery of surgical patients.^[4]^ Effective medical pain management would increase patients’ ability for physiotherapy and pulmonary rehabilitation which could sequentially improve postoperative outcome. Moreover effective medical pain management can reduce morbidity, shorten the length of stay and raised the patients’ satisfaction. A number of studies have confirmed the accurate analgesic effect of TPVB in thoracoscopic surgery.^[5–7]^ Therefore, it is an advantageous option for postoperative recovery after lung surgery. This study aims to evaluate the influence of ultrasound-guided thoracic paraspinal nerve block on perioperative pain and postoperative delirium in elderly patients undergoing VATS.

## 2. Materials and methods

### 2.1. Subjects

The study designed in a randomized, prospective, single-blind manner. And our study was approved by the Medicine Clinical Researches Ethics Committee of The Fourth Hospital of Hebei Medical University (2021012-1). Patients undergoing VATS above 60 to 80 years old with American Society of Anesthesiologists (ASA) classification 1 to 3 were included in the study. All patients were given written informed consent. Furthermore we screened all participant medical records. Patients excluded from participation were those identified as having: chronic pain and opioid using, local anesthetic allergy, spinal deformity, communication difficulties, psychiatric disorder, and Mini-mental State Examination < 25.^[8]^ Removing criteria: individuals with serious intraoperative complications (such as serious intraoperative cardio-cerebrovascular accidents), or individuals with serious postoperative complications resulting in prolonged hospital stay (such as postoperative bleeding requiring a second operation or postoperative transfer to ICU, etc.).

Individuals were enrolled according to analgesia regimen as group C or group T respectively. Randomization was achieved by computer generated random numbers and concealed using opaque closed envelopes.

### 2.2. General anesthesia

Upon arrival at the operating room, patients were monitored with 5-lead ECG, pulse oximetry (SpO_2_), invasive blood pressure (IBP), end-tidal carbon dioxide and BIS as standard ASA monitoring. Anesthesia induction was the same for all groups with propofol (1.5–2 mg/kg), sufentanyl (0.5–1 µg/kg) and double lumen tube (DLT) insertion facilitated with cisatracurium (0.2 mg/kg). The left position of DLT was confirmed with a fiberoptic bronchoscope. Anesthesia maintenance was achieved with sevoflurane in air-oxygen mixture and remifentanil (0.1–0.2 µg/kg/min), cisatracurium was administered intermittently to maintain muscle relaxation, to maintain the BIS index between 45 and 55. All patients received 1 to 2 µg/kg additional doses of fentanyl per hour and 0.1 mg/kg loading dose of morphine prior to thoracic closure. Perioperative adverse events (hypotension, bradycardia) were recorded.

### 2.3. Thoracic paravertebral block

After induction, anesthesiologist performed unilateral, single injection TPVB at T5 level with ultrasonography (GE Healthcare®, Wisguidance, Wauwatosa, WI) in group T. A high-frequency linear ultrasonography (probe frequency 7.5–10 MHz, depth 4.5–5.5 cm) was placed perpendicular to the dorsal midline on the target spinous process. The probe was moved to the affected side so that the screen showed both the target spinous process and the transverse process of the next stage. Move the probe slightly to the cephalic side to avoid the next stage of the transverse process, that is, the probe is located between and parallel to the two transverse processes, and the gap between the deep and lateral facet (about 1 cm) and the pleura is the paraspinal thoracic space. Peripheral block needle (Stimuplex® A; B Braun, Melsungen, Germany) was inserted from the outside of the probe, avoiding the pleura, using in-plane technique. The needle was placed in the paraspinal thoracic space during injecting 20 mL 0.375% ropivacaine to ensure that the pleura was moved down by the drug solution, indicating drug diffusion in the intervertebral space. Local anesthetic distribution above pleura was checked by moving the probe up and down to confirm.^[9–11]^ After the operation, patients’ vital signs, tidal volume, airway pressure, hipoxemia events and other respiratory parameters were observed.

### 2.4. Postoperative analgesia

All patients received postoperative patient-controlled intravenous analgesia. And the formula was as follows: dexocine 1 mg/kg and ketorolac ambutritol 3 mg/kg, diluted to 100 mL saline. If the VAS score ≥ 4 points at rest after the operation, the patient should carry out relief analgesia. After the exclusion of contraindications, flurbiprofen 50 mg or tramadol 100 mg can be given intravenously. Opioid analgesics would be used in case of unsuppressed pain.

### 2.5. Observational index

During the operation, anesthesiologists recorded the total amount of opioids, hipoxemia event, operative time, and time of offline extubation. A case of hipoxemia event is considered to have occurred as follow: the patient’s SPO_2_ have been lower than 90%, even the FiO_2_ is adjusted to 100%. VAS pain scores were recorded at 2, 4, 6, 24, 48, 72, 96, and 120 hours after operation by professionals (who did not participate in the administration of anesthesia). Visual analog score: 0 indicates no pain, 10 indicates most pain, and participants marked one of the 11 numbers according to their personal pain experience. Meanwhile evaluate the occurrence of postoperative delirium according to the nursing delirium screening score (Table 3).^[12]^ Each symptom is recorded as 0 to 2 points according to its severity, the highest score is 10 points, the total score of 2 points would be diagnosed as postoperative delirium.

**Table 3:** Nursing delirium screening score (Nu-DESC).

Symptom	Symptoms rating (0–2)	Score
I. Disorientation	Verbal or behavioral manifestation of not being oriented to time or place or misperceiving persons in the environment.	
II. Inappropriate behavior	Behaviour inappropriate to place and/or for the person; e.g., pulling at tubes or dressings, attempting to get out of bed when that is contraindicated, and the like.	
III. Inappropriate communication	Communication inappropriate to place and/or for the person; e.g., incoherence, noncommunicativeness, nonsensical or unintelligible speech.	
IV. Illusions/hallucinations	Seeing or hearing things that are not there; distortions of visual objects.	
V. Psychomotor retardation	Delayed responsiveness, few or no spontaneous actions/words; e.g., when the patient is prodded, reaction is deferred and/or the patient is unarousable.	
Total score		

Number of rescue analgesia, total amount of analgesic drugs, length of hospital stays and postoperative complications such as pneumonia or atelectasis within 5 days after operation were analyzed.

### 2.6. Statistical analysis

Statistical analysis were performed by SPSS 21.0 software. Measurement data were expressed as mean “*X ± S*” standard deviation, and comparison between groups was analyzed by *t* test. Chi-square test was used for counting data. *P* < .05 was considered statistically significant.

## 3. Results

A total of 211 participants were included in this study, among which 2 cases in group C were excluded due to excessive bleeding, and 1 case in group T was excluded due to severe postoperative pneumonia and transferred to ICU for treatment. Finally 105 cases in group C and 103 cases in group T were included in the research.

There were no significant differences in gender, ages, body weight, Mini-mental State Examination, preoperative hypertension or diabetes, BMI and ASA grade between two groups (Table 4).

**Table 4:** Demographic characteristics of patients and controls subjects.

	Group C (n = 105)	Group T (n = 103)	*P* value
Gender (M/F)	67/38	59/44	.395
Age (yr)	69 ± 4	68 ± 5	.650
Weight (kg)	68 ± 4	69 ± 4	.143
ASA (I/II)	40/65	38/75	.572
MMSE	28.6 ± 1.1	28.7 ± 1.1	.342
Preoperative hypertension	58/105	55/103	.790
Preoperative diabetes	36/105	42/103	.334

Values are expressed as means ± SD, or numbers (%).

ASA = American Society of Anesthesiologists, MMSE = Mini-mental State Examination.

Compared with group C, the intraoperative dosage of sufentanil and remifentanil in group T was significantly reduced (Table 1). The postoperative recovery time of the T group was shortened significantly, but there was no difference in cases of hipoxemia events nor the operative time between two groups. (Table 5).

**Table 5:** Total operating time and recovery time.

	Group C (n = 105)	Group T (n = 103)	*P* value
Operating time (min)	105 ± 18	100 ± 17	.056
Recovery time (min)	20 ± 6	16 ± 5**	<.01
Hypoxemia events (n)	8	10	.630

Values are expressed as means ± SD.

*P < .05.

** *P* < .01.

Compared with group C, VAS pain scores (static and dynamic) at 2, 4, 6, and 24 hours in group T were statistically significant. But there were no differences in VAS pain scores at subsequent time points (Tables 2 and 6).

**Table 6:** Postoperative dynamic VAS pain score.

	2 h	4 h	6 h	24 h	48 h	72 h	96 h	120 h
Group C (n = 105)	4.0 ± 1.2	4.0 ± 1.1	3.2 ± 1.0	3.0 ± 1.4	1.6 ± 0.6	0.7 ± 0.6	0.2 ± 0.4	0.04 ± 0.19
Group T (n = 103)	2.9 ± 1.0**	3.0 ± 1.0**	2.3 ± 0.7**	1.8 ± 0.6**	1.41 ± 0.8	0.7 ± 0.7	0.1 ± 0.3	0.05 ± 0.22
*P*	<.01	<.01	<.01	<.01	.088	.787	.165	.713

*P < .05.

** *P* < .01.

Compared with group C, the number of postoperative rescue analgesia, postoperative opioids consumption and the incidence of postoperative delirium was significantly decreased in group T. There was a deceased number of postoperative nausea, vomiting and postoperative complications, but no statistical significance between the two groups (Table 7).

**Table 7:** Postoperative general situation statistics.

	Number of remedial analgesia	Opioids consumption	Nausea and vomiting	PPCs	Delirium
Group C (n = 105)	3.8 ± 1.0	17 ± 4	40/105	10/105	28/105
Group T (n = 103)	2.1 ± 1.0**	9 ± 6**	32/103	8/103	13/103*
*P*	<.01	<.01	.31	.806	.014

PPCs = postoperative complications.

* *P* < .05.

** *P* < .01.

## 4. Discussion

Patients with ultrasound-guided TPVB had significantly reduced amount of postoperative analgesic drugs, shortened recovery time after surgery, enhanced postoperative analgesia, and reduced incidence of postoperative delirium. The results of this study demonstrate the exact analgesic effect of TPVB in thoracoscopic surgery. Several studies^[6–9]^ have shown that TPVB has a definite analgesic effect on thoracoscopy and thoracotomy. Due to the participants being aged 20 to 65 years old, a study suggests that ultrasound-guided nerve block has no effect on the awakening quality of patients undergoing thoracoscopic surgery.^[5]^ In this study, only elderly patients were selected, which may have led to inconsistent conclusions.^[13]^

The Enhanced recovery after surgery guidelines strongly recommend that all patients immediately start delusion screening after entering the post-anesthesia-care-unit until 5 days after surgery. And effective delusion scores for postoperative delusion screening should be accomplished.^[14]^ Once delirium was diagnosed, treatment should be initiated immediately. The later the treatment, the longer the duration of delirium and the higher the incidence of cognitive impairment. Therefore, in this study, the screening score of nursing delusion was recorded at 2, 4, 4, 6, 24, 48, 72, 96, and 120 hours after the operation (Table 6). Some researches have shown that there are many risk factors for postoperative delirium, which can be divided into two categories: susceptible factors and inducing factors.^[15]^ Susceptibility factors mainly refer to the risk factors that exist before admission, such as advanced age, cognitive impairment, preoperative anemia, low ejection fraction, carotid artery stenosis, high creatinine level, multiple organ dysfunction, visual impairment, hearing impairment, and alcoholism. Risk factors include pain, co-infection, limited activity (protective restraint), hypoxemia, hydroelectrolyte disorder, acid-base imbalance, urinary retention, constipation and sleep deprivation, etc. Among them, three factors are strongly suggested to be valued in clinical work: cognitive loss associated with combined alcohol, prolonged surgery, and postoperative pain. Studies have also shown that pain stimulation cause hyperfunction of the hypothalamic-pituitary-adrenal cortical axis, increase the release of glucocorticoids and inflammatory cytokines, activate the central nervous system and brain hippocampus, thus increase the risk of mental disorders and cognitive dysfunction.^[16–18]^ In our study which is not consistent with the conclusion of Lee’s study,^[5]^ the participants were elderly patients who were sensitive to the anesthetic drugs.^[19]^ Because of the good analgesic effect provided by TPVB the total amount of intraoperative anesthetic drugs was decreased. Thus reduction of recovery time after surgery is logical. Similarly, the good analgesic effect provided by TPVB also significantly reduced postoperative pain, manifested as the reduction of VAS score both static and dynamic within 24 hours after surgery, the less number of postoperative rescue analgesia and the decreased total amount of analgesic drugs. So the incidence of postoperative delirium was significantly lower in elderly patients in the T group. There was no difference in the incidence of intraoperative hypoxemia between the two groups, suggesting that hypoxmia events had not influenced the results in our study. However, there was no significant difference in the incidence of postoperative nausea and vomiting and postoperative pulmonary complications between the two groups, which was unexpected. Perhaps, the reason is: our sample size was not large enough, and the preoperative pulmonary and different surgical procedures were not analyzed.

It is undeniable that there are still some limitations in our present study. We did not collect data regarding the motor subtypes of delirium, the number of delirium episodes. Due to the limitations of technical personnel, a more sensitive but burdensome and complex scale, S-PTD, was not used.^[20]^ Preoperative pulmonary and other surgical procedures should be analyzed. This was a single-center study, and the sample size was not sufficiently large. A large-sample, multicenter, multifactorial randomized trial is needed to further clarify the role of postoperative delirium.

In conclusion, preoperative ultrasound-guided TPVB analgesia can reduce the amount of intraoperative analgesic drugs in elderly patients undergoing thoracoscopic lobectomy, shorten the awakening time, reduce the number of postoperative rescue analgesia and reduce the incidence of postoperative delirium.

## Acknowledgments

The authors would like to thank all the members of Department of Anesthesiology, Fourth Hospital of Hebei Medical University for their great help and support.

## Author contributions

**Conceptualization:** Qiu Dongjie, Zhao Longbiao, Liu Peng.

**Data curation:** Qiu Dongjie, Chang Zhiyan, Yu Long.

**Formal analysis:** Qiu Dongjie, Liu Peng.

**Funding acquisition:** Qiu Dongjie.

**Resources:** Jia Li, Xu Hongmeng.

**Software:** Qiu Dongjie.

**Writing – original draft:** Qiu Dongjie.

**Writing – review & editing:** Zhao Longbiao, Xu Hongmeng.
